# Taxonomic Composition and Salinity Tolerance of Macrozoobenthos in Small Rivers of the Southern Arid Zone of the East European Plain

**DOI:** 10.3390/biology12091271

**Published:** 2023-09-21

**Authors:** Larisa V. Golovatyuk, Larisa B. Nazarova, Irina J. Kalioujnaia, Ivan M. Grekov

**Affiliations:** 1 Papanin Institute for Biology of Inland Waters, Russian Academy of Sciences, Borok, Nekouzsky District, 152742 Yaroslavl Oblast, Russia; 2 Institute of Ecology of the Volga River Basin, Samara Federal Research Scientific Center, Russian Academy of Sciences, Komzina Str. 10, 445003 Tolyatti, Russia; 3 Institute of Geology and Petroleum Technologies, Kazan Federal University, Kremlyovskaya Str. 18, 420008 Kazan, Russia; nazarova_larisa@mail.ru; 4 Alfred Wegener Institute, Helmholtz Centre for Polar and Marine Research, Research Unit Potsdam, Telegrafenberg A43, 14473 Potsdam, Germany; 5 Faculty of Geography, Lomonosov Moscow State University, Leninskie Gory 1, GSP-1, 119991 Moscow, Russia; kalioujnaia@yandex.ru; 6 Faculty of Geography, Herzen State Pedagogical University, Moika 48, 191186 St. Petersburg, Russia; ivanmihgrekov@gmail.com

**Keywords:** benthic communities, species composition, species distribution, salinity resistance, salinity gradient, small rivers, arid region, East European Plain, the Volga River, the Lake Elton

## Abstract

**Simple Summary:**

Climate-related salinization of inland waters is observed in many regions of the world as a major environmental problem affecting natural processes in aquatic ecosystems. In order to better predict and control these changes, it is important to study the responses of aquatic fauna to increasing salinity. Macrozoobenthic fauna, which includes mollusks, small crustaceans, and insect larvae, constitutes the main food base for fish and water birds. Due to their relatively short life cycles, large species diversity, and high abundance, macrozoobenthos are the best indicators of changing water salinity. To determine the species richness, distribution, and salinity tolerance of macrozoobenthos, we investigated 17 small rivers with different water salinity in the southern arid region of the East European Plain. The study shows that the species richness gradually decreases with an increase in water salinity in the rivers. In freshwater rivers, the macrozoobenthos fauna includes more than 100 species, whereas, in hypersaline rivers with salinity comparable to seawater, only 10 species were found. A total of 5 of the 156 invertebrate species can be used as indicators of water salinization in rivers of the arid regions of Europe.

**Abstract:**

This study investigated the species composition, distribution, and salinity tolerance of macrozoobenthos in 17 small rivers in the southern arid region of the East European Plain, which are characterized by a small channel gradient, slow-flowing or stagnant water bodies, and a wide range of water salinity, varying between 0.18 and 30 g L^−1^. In total, 156 taxa were found, among which 66 were Diptera species. The study revealed that the formation of benthic communities in the rivers is influenced by natural factors of the catchment basins, including the flat landscape with sparsely developed relief differentiation, climate aridity, and the widespread occurrence of saline soils and groundwater, largely related to the sedimentation of the ancient Caspian Sea and modern climate changes. These conditions are favorable for the occurrence of lacustrine macrozoobenthic species in freshwater, euryhaline, and halophilic ecological groups. The investigation revealed a decrease in species richness in response to an increase in water salinity. The five identified halophilic species *Tanytarsus kharaensis*, *Glyptotendipes salinus*, *Cricotopus salinophilus*, *Chironomus salinarius*, and *Palpomyia schmidti* can be used as indicators of river ecosystem salinization.

## 1. Introduction

An increase in the salinity of inland waters is observed in many regions of the world and expands globally [1,2]. Changes in salinity can be induced by climate warming and by increasing anthropogenic impact [3,4,5,6], degrading the stability of the natural environment and species diversity across the globe [7,8]. Salinization of natural waters is one of the main factors causing the disruption of the normal functioning of rivers in the world and is as harmful as pollution by pesticides [9].

The identification of halotolerant and halophilic species helps reveal differences between the faunas of freshwater and saline water bodies [10]. To predict possible changes in freshwater ecosystems under the influence of increasing environmental hazards and to identify “early warning ecosystem signals”, it is extremely important to study biotic communities across a wide range of environmental gradients, including salinity [11,12,13,14].

The vast area of the Volga River basin, part of which was influenced by transgressions and regressions of the ancient Caspian Sea [15,16], is among the areas at high risk of salinization of aquatic water bodies, strengthened by the location of the lower Volga River basin in the arid climate zone. In general, climate change observations across Russia over the past 50 years confirm an increase in the average annual precipitation (2.2% per 10 years), an increase in the total annual surface runoff (by 200 km^3^), and a steady increase in the average annual air temperature (by 0.51 °C per 10 years) [3,17,18]. However, to the south of the European part of Russia, the effects of climate change differ from the observed averages for Russia [19,20,21]. In particular, in the Volga River basin, observations show a more significant increase in the average annual air temperature up to 2.2 °C during the past 60 years or a mean of 0.036 °C per year [20,22], a decrease in water discharge by 40–60% [3,19,23,24], and a fluctuation in the moisture supply from a maximum between 1980 and 1994 to minimum values since 2000 [20]. These changes lead to annual droughts, a lowering of local and regional groundwater levels, shallowing and even drying up of water bodies, and an increase in the total salinity of waters, which negatively affect all components of terrestrial and aquatic ecosystems [4,6,18,25,26].

Small rivers in the arid zone of the East European Plain are characterized by significant differences in salinity [26,27,28,29,30]. At the same time, the biotic communities of these rivers, which serve as indicators of the ecological state of aquatic ecosystems, have so far been studied only fragmentarily. A comprehensive investigation was conducted only at the beginning of the 20th century [31], while more recent studies in the area are related to the collection and identification of individual groups of aquatic organisms only [32,33,34,35].

Therefore, the main aims of our investigation are to study the species composition and distribution of macrozoobenthos in a large selection of small rivers in the southern arid zone of the East European Plain, to identify specific euryhaline and halophilic species, and to analyze the relationship between the taxonomic richness of macrozoobenthos and the level of surface water salinity.

## 2. Materials and Methods

### 2.1. Study Area

**Figure 1:**
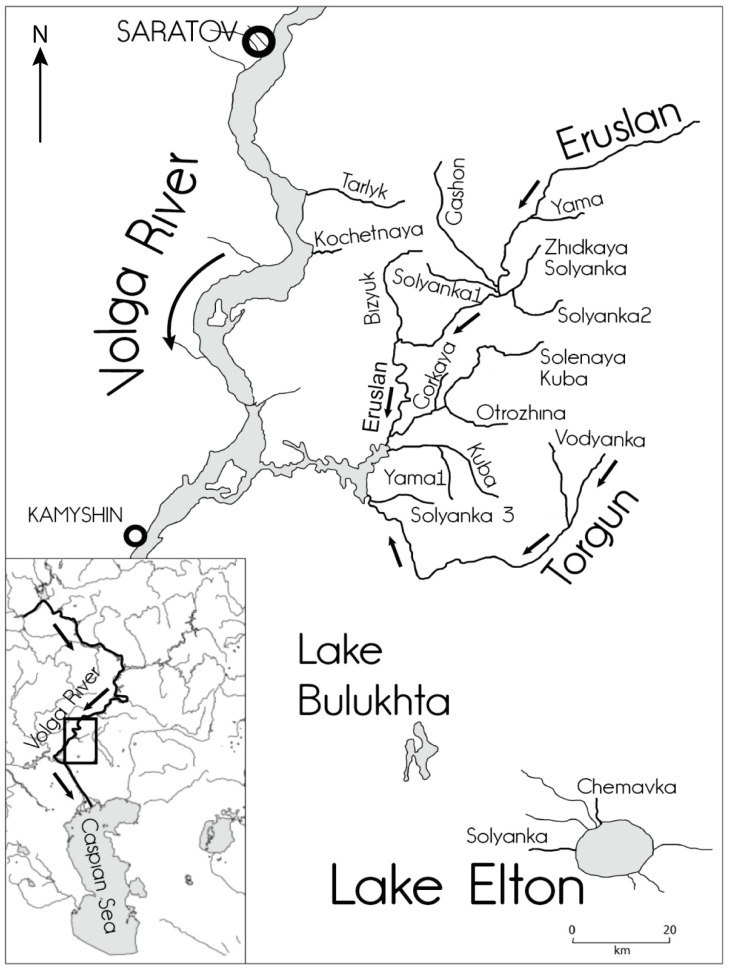
Study area and location of the investigated small rivers.

The investigation focused on small rivers in the southern part of the East European Plain between 49°00′–51°21′ N and 45°45′–47°48′ E in the ecotone zone between the Pontic dry steppes and the Turan semi-deserts [27,36] (Figure 1). The territory is rather pristine and scarcely populated (5–10 people/km^2^) without large settlements [17,37,38,39].

The climate of the study area is severely continental, with long hot dry summers, cold dry winters, a high amplitude of average and extreme monthly air temperatures, a strong moisture deficit (Aridity Index 0.3–0.5), and frequent strong winds and droughts [17,18,19,20,21,27,40]. The mean annual air temperature is 6 to 8 °C, increasing in a southerly direction. The mean July air temperature (the warmest month) varies from 22.9 °C in the north to 25.7 °C in the south, whereas the mean February air temperature (the coldest month) varies from −9.7 °C to −7.0 °C. The maximum summer temperature can reach +45 °C in July or August, whereas the lowest winter temperature can drop to −40.7 °C in January or February. From north to south, the annual precipitation declines from 380 to 270 mm per year, and the open water evaporation rate increases from 800 to 850 mm [17,18,37,38,39,40].

The northern part of the study area is characterized by sloping lacustrine-alluvial, ancient alluvial, and loess-type watershed plains with absolute heights declining from 100 to 50 m in some places that are ridged and poorly dissected by watercourses [15,17,27,37,38]. The small rivers in this area are typical lowland small rivers that are part of the left-bank tributary network of the Volga River basin [41]. The majority of the small rivers are second-order tributaries of the Volga River (Table 1), with only two small rivers being first-order tributaries (the Tarlyk and the Kochetnaya) and one small river (the Solyanka-2) being a third-order tributary of the Volga River.

The southern part of the study area belongs to the Lake Elton basin and is characterized by low-lying (10 to 35 m a.s.l.), almost flat, marine accumulative sand-clay and clay saline plains, with drainless depressions and salt lakes formed under the influence of transgressions and regressions of the ancient Caspian Sea [15,16,17,37,39]. The small rivers in this area included in the investigation—the Chernavka and the Solyanka rivers—are first-order tributaries of the hyperhaline Lake Elton, the largest closed drainage depression [42,43,44,45].

### 2.2. Characteristics of Watercourses

Across the study area, the hydrographic network is rather poor. All selected 17 small rivers (Table 1) have meandering channels with occasionally steep banks (up to 10 m) and fluvial terraces. In the lower reaches, the rivers are rather broad (50 to 120 m in width). The depth of the channels varies from several tens of cm on the riffles to 1–3 m on the reaches. The tributaries in the Lake Elton basin are characterized by a higher gradient of stream slope (up to 5.5‰) and faster stream velocity (0.01–0.09 m s^−1^) than the tributaries to the Volga River basin (up to 2‰ and 0.02–0.4 m s^−1^ accordingly), caused by active extensions of halotectonics.

The investigated rivers are fed mainly by snow-melt water. Up to 70% of the runoff occurs during a short period of spring flood, characterized by a sharp rise and decline in discharge, and, accordingly, water levels. During the flood peak in May, the water level rises by 1 to 5 m. During the period of low discharge, the rivers become very shallow, often breaking into separate reaches with an almost complete absence of flow towards the end of summer. In autumn, with an increase in precipitation, the runoff increases slightly. The rivers freeze mainly during the second half of November and open in early April [26,28,38,39,41].

The salinity and chemical composition of the river water are highly correlated with geographical zonality. A general north–south trend of increasing salinity (from 0.18 to 30 g L^−1^) can be observed, while the water composition changes from calcium hydrocarbonate to sodium chloride [17,41]. The water salinity and morphometric characteristics of the studied small rivers and their basins are presented in Table 1. The data were obtained from our own hydro-chemical studies [44], augmented by information extracted from the State Water Register [46] and relevant publications [26,28,41].

Further characteristic features of the 17 rivers studied are also valid for the majority of other rivers in the arid part of the East European Plain [26,28,29,30], including a regulation of water flow by permanent or temporary dams and an intensive overgrowth by macrophytes. The most common river sediment texture includes silt, clay or silt, clay, sand, and plant detritus.

### 2.3. Field Sampling

Sampling of macrozoobenthos in the hypohaline, oligohaline, and mesohaline tributaries of the Volga River was studied during the summer seasons of 2015 to 2017, and in the polyhaline tributaries of the Lake Elton basin during the summer seasons of 2017, 2018, and 2023. Sediment samples were collected from the river channels and the shores in the upper, middle, and lower reaches of the rivers. The sampling sites were selected on river stretches with a natural flow regime to exclude the possible backwater impacts of permanent or temporary reservoirs on the composition of macrozoobenthic communities. At each sampling site, samples were taken using an Ekman-type grab sampler (25 cm^2^) and/or a handle-blade trawl (pulling 0.5 m). Due to the small sampling area, eight grab replicates were pooled together into one sample immediately after material collection. All samples were washed through a nylon sieve (mesh size 300–333 μm) and fixed with a 4% formaldehyde solution. In total, 120 samples from 50 sampling locations were collected and processed.

At each river site, we used field analytical instruments for measuring pH (HANNA pH Tester HI 98127, HANNA Instruments Deutschland GmbH, Vöhringen, Germany), oxygen content (HANNA Oximeter HI 9146, HANNA Instruments Deutschland GmbH, Vöhringen, Germany), and current velocity (ISP-1, Hydrometeopribor LLC, St. Petersburg, Russia).

### 2.4. Species Identification

Laboratory processing of the samples, subsequent microscopy, and identification of aquatic organisms were carried out according to standard methods [47]. Macrozoobenthic species were identified using widely accepted identification guides updated to include recent taxonomic revisions [48,49,50,51,52,53,54,55], others, and up-to-date online databases [56,57].

### 2.5. Data Analyses

The water salinity of the samples collected was measured at the Center for Monitoring of Water and Geological Environment in Samara, Russian Federation. Salinity classes were determined in accordance with the Venice salinity classification [58]: freshwater or hypohaline (<0.5 g L^−1^), oligohaline (0.5–5.0 g L^−1^), mesohaline (5–18 g L^−1^), and polyhaline (18–30 g L^−1^).

The distribution of species in the rivers was analyzed using the calculated frequency of occurrence (F, %) of species across all samples [47].

The data set was analyzed to examine the relationship between the environmental variables and the distribution and abundance of macrozoobenthic organisms. All taxa data were transformed to percent abundances, calculated as the percentage of total identifiable specimens [59,60], and were square root transformed before analysis. Environmental variables were controlled for skewness, and variables with skewed distributions (current velocity and catchment area) were log-transformed. Skewness reflected the degree of asymmetry in the distribution around the mean. Normal distributions produced a skewness statistic of about zero. Values that exceeded two standard errors of skewness (regardless of signs) were identified as significantly skewed [61]. The remaining parameters were left untransformed.

Detrended Correspondence Analysis (DCA) with detrending by segments was performed on the macrozoobenthos data (rare taxa downweighted) to explore the main pattern of taxonomic variation among sites and to determine the lengths of the sampled environmental gradients, from which we decided whether unimodal or linear statistical techniques would be the most appropriate for the data analysis [62]. The gradient length of the species score was relatively long. DCA axes 1 and 2 were 7.964 and 2.269 standard deviation units, respectively, indicating that numerical methods based on a unimodal response model were the most appropriate to assess the variation structure of the chironomid assemblages [63].

Variance inflation factors (VIF) were used to identify intercorrelated variables. Environmental variables with a VIF greater than 20 were eliminated, beginning with the variable with the largest inflation factor, until all the remaining variables had values < 20 [64].

Relationships between macrozoobenthos distribution and environmental variables were assessed using a set of Canonical Correspondence Analyses (CCA), with each environmental variable as the sole constraining variable. The percentage of the variance explained by each variable was calculated. The statistical significance of each variable was tested using a Monte Carlo permutation test with 999 unrestricted permutations [65]. Significant variables (*p* ≤ 0.05) were retained for further analysis. Both DCA and CCA were performed using CANOCO 4.5 [64].

## 3. Results

### 3.1. Fauna Structure and Species Richness

In all samples collected from the investigated small rivers, a total of 156 benthic taxa were identified, including 66 species of Diptera, 16 species of Oligochaeta, 16 species of Coleoptera, 15 species of Mollusca, 11 species of Heteroptera, 8 species of Crustacea, 8 species of Odonata, 7 species of Trichoptera, 3 species of Ephemeroptera, 4 species of Hirudinea, one species of Megaloptera, and 1 species of Lepidoptera (Table 2). The total number of species observed in individual samples from small rivers varied from a minimum of 6 species in the Solyanka River up to a maximum of 72 in the Solenaya Kuba River (Table 3). Chironomid larvae and oligochaetes were permanent components of the fauna in all small rivers.

### 3.2. Distribution of Taxonomic Groups

The oligochaetes *Limnodrilus hoffmeisteri* (F = 73%), and the chironomids *Polypedilum nubeculosum* (F = 58%) and *Chironomus plumosus* (F = 49%) were most frequently found in the bottom communities of the hypohaline, oligohaline, and mesohaline tributaries of the Volga River.

In these small rivers, where significant areas of the river bottom are occupied by aquatic vegetation, the subclass Oligochaeta included eight species from the subfamily Naidinae, six species from the subfamily Tubificinae, and one species was recorded from the families Lumbriculidae and Enchytraeidae. The oligochaetes *Tubifex tubifex* was noted in almost half of the samples, whereas the species *Nais barbata* (the Zhidkaya Solyanka River), *N. communis* (the Gorkaya River), and *N. pseudobtusa* and *Uncinais uncinata* (the Tarlyk River) were rare in the investigated rivers.

The frequency of the occurrence of leeches did not exceed 6%. Leeches were represented by species widely distributed in the medium and small rivers of the Volga River basin and were found among the macrophytes along the streambanks and in silted grounds in different sections of the studied small rivers.

All bivalve species typically observed in small rivers [66] were rare in the sampled macrozoobenthic communities; only four species were discovered, each with a single occurrence. The gastropods had a wider distribution, with the highest species richness found within the genus Lymnaea.

Among crustaceans, the majority of species (76%) belonged to alien fauna, which was only found in the mouth areas of the Kochetnaya and Tarlyk rivers. Of the native crustaceans, *Asellus aquaticus* (15%) was a permanent resident in almost all investigated small rivers inhabiting different parts of the studied lotic ecosystems.

Mayfly larvae were found in almost all rivers, except for three rivers—the Gorkaya, Solyanka 1, and Solyanka. Ephemeroptera included only three species from the families Caenidae and Baetidae and were found in macrophyte thickets. Mayflies *Caenis robusta* (F = 25%) and *Cloeon simile* (F = 14%) occurred in our samples frequently, whereas *C*. gr. *dipterum* was quite rare (F = 2%). No clear confinement of mayfly larvae to certain sections of the studied rivers was observed.

Phytophilic representatives of caddisflies from the *Hydroptilidae*, *Leptoceridae*, *Polycentropodidae*, and *Phryganeidae* families were collected from streambank thickets of sedge, pondweed, and hornwort. The frequency of caddisfly species occurrence did not exceed 6% (*Oecetis furva* and *Ecnomus tenellus*).

Dragonfly larvae showed high diversity and were mainly found in the overgrown areas of the rivers. Among dragonflies, the species *Sympecma fusca* and *Enallagma cyathigerum* were the most common (7% and 5%, respectively).

The orders Coleoptera and Heteroptera did not include highly specialized species or rheophilic forms. All taxa were typical representatives of the limnophilic fauna. The larvae of beetles *Haliplus* sp. and *Laccophilus* sp., and bugs *Plea minutissima* and *Ilyocoris cimicoides* were found in the rivers with the highest frequency.

Chironomid larvae were a permanent component of the Diptera fauna in all the rivers. With 41 taxa, the subfamily Chironominae showed the greatest taxonomic richness. In the subfamily Orthocladiinae, 13 species were recorded, while the Tanypodinae subfamily included 10 species. The majority of chironomid species belonged to limnophilic or eurybiontic fauna: Tanypodinae *Procladius ferrugineus* (36%), *Tanypus punctipennis* (33%), Orthocladiinae *Cricotopus* gr. *sylvestris* (41%), *Psectrocladius sordidellus* (25%), Chironominae *Polypedilum nubeculosum* (58%), and *Chironomus plumosus* (49%). The most widespread was *Sphaeromias pictus* (24%) from the order Diptera family Ceratopogonidae.

In contrast to the macrozoobenthic fauna of the studied 15 hypo-, oligo- and mesohaline rivers of the Volga River basin, the taxonomic composition of the polyhaline Solyanka and Chernavka rivers of the Lake Elton basin was very poor (six and eight species, respectively). Mayflies, caddisflies, leeches, crustaceans, and dragonflies were not recorded in these streams. The benthic communities were composed only of Diptera, Coleoptera, and Heteroptera larvae (Table 2). The Chironomids *Cricotopus salinophilus* (100%), *Chironomus salinarius* (30%), and Ceratopogonidae *Palpomyia schmidti* (30%) had the highest frequency of occurrence in the polyhaline rivers.

### 3.3. Benthic Assemblages in Rivers of Different Salinity

**Figure 2:**
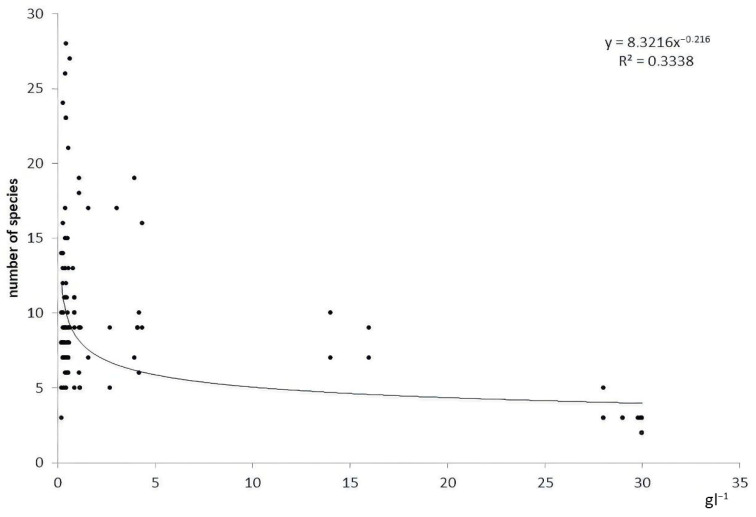
Relationship between species richness and salinity of the rivers.

We observed that the taxonomic richness of macrozoobenthos was much higher in river stretches with lower salinity than in river stretches with higher salinity (Figure 2).

**Figure 3:**
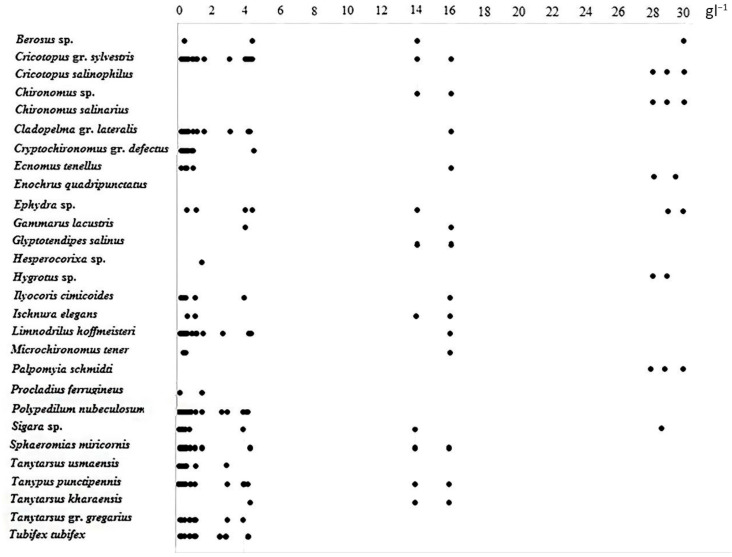
Distribution of the most common benthic taxa in the investigated small rivers in relation to salinity gradient.

The majority of all recorded taxa of macrozoobenthos (128 species, or 82%) were found only in hypohaline and oligohaline waters within the range of water salinity from 0.18 to 4.34 g L^−1^. A total of 28 or 18% of all recorded taxa were more tolerant to salinity levels and were distributed in a wider range of salinity from the hypohaline to mesohaline (16 g L^−1^) or polyhaline waters (28–30 g L^−1^) (Figure 3).

**Figure 4:**
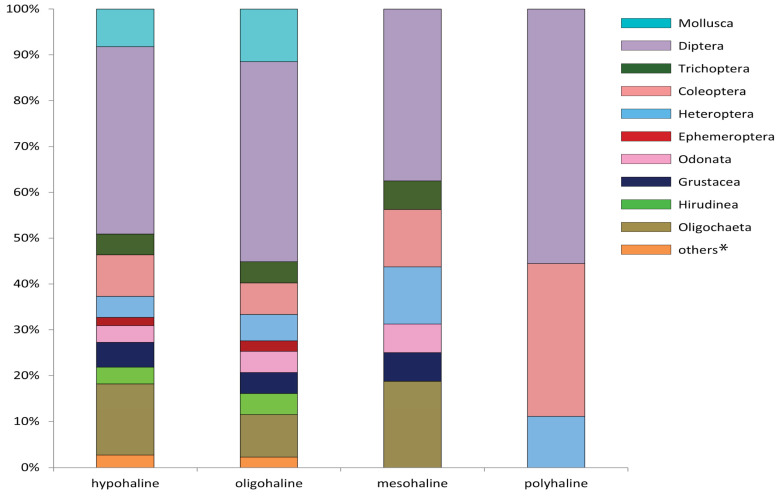
Summarized species richness of macrotaxons for river sections with different salinity. * Others—Sialidae, Lepidoptera, and Aranai.

The analysis of the species richness for parts of rivers with different salinity levels revealed that the ratio of macrotaxons gradually changed with changes in salinity (Figure 4). The communities of hypohaline and oligohaline samples were more diverse and included 11 taxonomic groups, compared to 6 taxonomic groups in mesohaline samples and only 3 groups in polyhaline samples. Mayflies, leeches, and mollusks were not found in mesohaline sections of the rivers, whereas the fauna of polyhaline rivers did not include mayflies, caddisflies, leeches, crustaceans, mollusks, oligochaetes, or dragonflies. The order Diptera was the dominant taxa in all types of rivers, from hypohaline to polyhaline, whereas the proportion of Diptera larvae was the highest in polyhaline rivers, representing up to 56% of the benthic fauna. The main subdominant macrotaxons were Oligohaeta for hypohaline, oligohaline and mesohaline rivers, and Coleoptera for polyhaline rivers.

### 3.4. Relationships between Macrozoobenthos Distribution and Environmental Variables

CCA with all environmental parameters showed that salinity max, salinity min, salinity average, and the river parameters (stream order, catchment area, average stream slope, length, and current velocity) were intercorrelated and were removed from the analysis one by one until all VIFs were below 20. A minimal subset of uncorrelated environmental parameters included salinity average, pH, and O_2_. Monte Carlo test (999 permutations) showed that all these parameters played a significant role in the macrozoobenthos distribution (*p* ≤ 0.05).

The eigenvalues of CCA axes 1 and 2 (λ1 = 0.891 and λ2 = 0.193) of the three significant variables constituted 99% and 88.5% of the eigenvalues of CCA axes 1 and 2 of the full set of the known environmental variables (Table 4), suggesting that the removal of correlated and insignificant variables had little impact on the effectiveness of the analysis. According to S. Juggins [67], the ratio of the eigenvalues of CCA axes 1 and 2 below 1 implies that not all the important environmental parameters were included in the analysis. In our case, the ratio was 4.62 (λ1/λ2 = 0.891/0.193), indicating that the most important parameters were included in the analysis.

**Figure 5:**
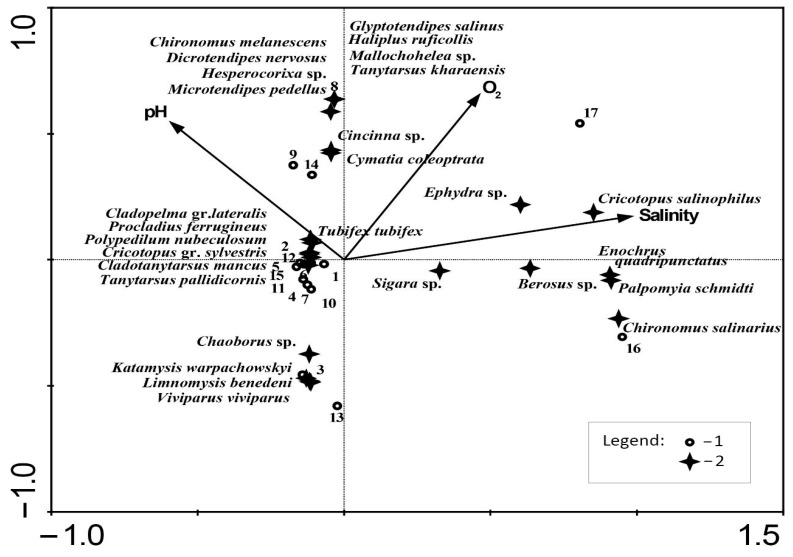
CCA triplot of the significant environmental variables, investigated rivers, and the most common macrozoobenthic taxa. Legend: 1–the investigated rivers numbered from 1 to 17 according to the list provided in Table 1; 2–the identified benthic taxa.

CCA axis 1 (Figure 5) is most strongly correlated with salinity. The polyhaline Cernavka (16) and Solyanka (17) rivers are attributed to the right part of the triplot. Typical for this group of the rivers are the most tolerant to salinity taxa: *Chironomus salinarius*, *Cricotopus salinophilus*, *Palpomyia schmidti*, *Enochrus quadripunctatus*, *Berosus* sp., and *Ephydra* sp. The species *Chironomus salinarius*, *Palpomyia schmidti*, *Enochrus quadripunctatus,* and *Berosus* sp. demonstrate no significant relation to O_2_, whereas the distribution of *Cricotopus salinophilus* and *Ephydra* sp. is associated with O_2_.

The fauna of the Otrozhina River (8) is more related to the higher concentration of oxygen and higher pH. The river contains oligohaline in its upper reach and mesohaline in its lower reach. The species attributed to this river include halophilic (*Glyptotendipes salinus* and *Tanytarsus kharaensis*) and freshwater species (*Microtendipes pedellus*, *Dicrotendipes nervosus*, and *Chironomus melanescens*).

The sites of the downstream sections of the Tarlyk (3) and Kochetnaya (13) rivers affected by the backwater formation of the Volgograd Reservoir are inhabited by the alien species *Katamysis warpachowskyi* and *Limnomysis benedeni*, which do not appear elsewhere in the investigated set of rivers.

The majority of sites of the investigated rivers (central part of the triplot) include species that demonstrate low tolerance to extreme values of the limiting factors. These are eurybiontic species (*Tubifex tubifex*, *Cladopelma* gr. *lateralis*, *Procladius ferrugineus*, *Polypedilum nubeculosum*, *Cymatia coleoptrata*, *Cincinna* sp., etc.) living in hypohaline and oligohaline sections of rivers.

## 4. Discussion

The relationship between salinity content and species composition of aquatic communities has been well investigated for salt lakes and estuaries [68], but only partially studied for rivers, including saline and hypersaline [69,70,71,72,73].

The current investigation presents for the first time a comprehensive and detailed analysis of macrozoobenthos in a large selection of 17 small rivers of the Volga River basin and the Lake Elton closed drainage basin in the southern arid region of the East European Plain, and their relationship to salinity.

In the early twentieth century, the Russian hydrobiologist A.L. Bening [31] collected the first scientific data on the faunal composition of the benthic communities of the studied hypohaline, oligohaline, and mesohaline small tributaries of the Volga River, identifying eight species of beetles, two species of caddisflies, and one species of alderflies in the Solenaya Kuba River. Later, V.V. Anikin and E.V. Ugolnikova [35] studied the dragonfly fauna of the region, identifying the images of five species from the Lestidae, Aeshnidae, and Libellulidae families in the Bizyuk River. O.G. Brekhov further investigated the fauna and ecology of various families of the order Coleoptera in the study area [32,33]. The low species diversity of benthic fauna of the polyhaline Chernavka and Solyanka rivers in 2003 was recorded in V.P. Gorelov [34] from 2008 to 2013 by L.V. Golovatyuk and V.K. Shitikov [44] and T.D. Zinchenko et al. [74].

Most of the 156 species recorded in the investigated small rivers represent species of benthic taxa widespread in the waterbodies of the European part of Russia [75,76,77]. A characteristic feature of the macrozoobenthic fauna is the dominance of limnophilic species. Stonefly larvae and other specifically rheophilic groups were not recorded in the benthic communities. Only a few species of Ephemeroptera and Trichoptera, which are taxa usually associated with flowing waters, were observed to occur in the study area.

Our results are consistent with the data obtained in the early 20th century [78]. It has been reported that the macrozoobenthic communities of the Solenaya Kuba River are dominated by the taxa characteristic of slow-flowing and stagnant water bodies. This supports our results and the hypothesis that the plain (flat) landscape structure of the region and the natural hydrological features of rivers in the arid zone play an important role in the formation of the fauna.

Our previous studies showed that there are significant differences in the fauna composition between small rivers in the arid (steppe, semi-desert) and forest-steppe zones of the Volga River basin [76]. Specifically, the macrozoobenthic communities of rivers in the semi-desert zone are depleted in comparison to rivers in the steppe and forest-steppe zones, primarily due to an increase in water salinity and lower stream gradients of semi-desert rivers [79].

The taxonomic richness of macrozoobenthos in small rivers belonging to the Volga River basin and Lake Elton closed drainage basin differed significantly. The majority (82%) of the taxa were recorded in the hypohaline and oligohaline rivers of the Volga River basin with water salinity up to 4.3 g L^−1^ and belonged to the freshwater and euryhaline ecological groups. Only a few species registered at a salinity level of 14–16 g L^−1^ fit into the halophilic ecological group. On the contrary, in the polyhaline rivers of the Lake Elton basin with a salinity of more than 28 g L^−1^, the taxonomic composition is poorer and includes only euryhaline and halophilic species typical of high-salinity rivers. For example, in the saline Rambla Salada River (Spain), only eight taxa were recorded at a salinity of ~100 g L^−1^ [73].

Our investigation of macrozoobenthos confirms that the structure of benthic communities is changing with increasing salinity. The number of taxonomic groups is gradually decreasing from hypohaline and oligohaline river sections of the Volga River basin to mesohaline sections in the same basin, while a minimum number of taxa is observed in polyhaline rivers of the Lake Elton basin, in which the total number of taxa is almost four times less than that in hypohaline and oligohaline rivers. The macrozoobenthic fauna of mesohaline river sections lacked representatives of mayflies, leeches, and mollusks, whereas caddisflies, crustaceans, oligochaetes, and dragonflies do not occur in addition to the above taxa in polyhaline river sections. The ratio of macrotaxons remains almost stable for the hypohaline and oligohaline river sections, which is compatible with the results obtained by S.D. Rundle et al., who studied the estuary of the Yealm River, UK [69], and by C. Piscart et al. [71,72], who studied the Meurthe River in northeastern France. At the same time, the communities of mesohaline river sections demonstrate a higher ratio of Heteroptera and Coleoptera, whereas the ratios of Diptera and Coleoptera were significantly higher in communities of polyhaline rivers.

Considering the wide range of salinity levels in the studied rivers, we also reviewed the well-known “the Remane’s principle” [80] and the related concept of “the critical salinity”. The salinity ranges from 5 to 8 g L^−1^, which is considered the zone of “critical salinity” (=horohalinicum) in which a “minimum of species” occurs [81,82], was not recorded in the studied rivers of the Volga River and Lake Elton basins. Nevertheless, the study revealed that hypohaline and oligohaline river sections with salinity levels up to 4.3 g L^−1^ are characterized by a significant diversity in macrozoobenthic fauna, with the absolute majority of freshwater species. In response to the salinity levels increasing up to 14-16 g L^−1^, the macrozoobenthic fauna is changing to include typical brackish-water species. Finally, the benthic communities of polyhaline river sections, in which salinity levels exceed 28 g L^−1^, are represented only by species typical of brackish and halophilic aquatic environments. Considering that the diversity of brackish-water species is limited all over the world [82], the notable decrease in species richness observed in the studied rivers with salinity levels up to and exceeding 14 g L^−1^, our findings are consistent with the fundamentals of the concept of critical salinity.

Our study of small rivers also confirmed that macrotaxons like Ephemeroptera, Hirudinea, and Mollusca are sensitive to increasing salinity, among which the order Ephemeroptera is often indicated as including the most sensitive taxa [71,72,83,84]. The study also confirmed that the species most resistant to high salinity levels belong to the taxa from the orders Diptera, Heteroptera, Coleoptera, Odonata, and Trichoptera. This is especially evident in the samples from the polyhaline Chernavka and Solyanka rivers, where macrozoobenthic taxa from the orders Diptera, Coleoptera, and Heteroptera constitute up to 100% of the fauna. The largest number of salinity-tolerant species was found in the family Chironomidae (Diptera). These observations are in line with observations of the predominance of Diptera and Coleoptera in benthic communities in highly saline sections of saline rivers in Spain [73,85], lakes in the USA [86] and in North Africa [87], which found that, among the order Diptera, the Ceratopogonidae species can survive salinity levels up to 108 g L^−1^, Ephydridae species up to 100 g L^−1^, and Chironomidae species up to 115 g L^−1^ [73,88,89]. The Coleoptera species found in the rivers of Spain and southwestern Australia occur in aquatic environments with salinity levels reaching up to 81–135 g L^−1^ [73,88,89,90,91].

For a number of species in these orders, salinity is not a limiting factor, which is explained by the evolutionary history of these species [13]. The ecological adaptations of Diptera to survive in extreme conditions include a short life cycle, high fertility of the imago, the ability to actively settle, greater mobility, and the use of the same substrate for food by larvae and imago [92]. In addition, a number of species of the Ephydridae family living in conditions of high salinity use cyanobacteria unused as food by other species of aquatic insects. This allows them to avoid competition for food, which increases their chances of surviving in extreme environments [93].

Twenty species from the studied small rivers, including caddisflies *Ecnomus tenellus*, can be attributed to euryhaline taxa, whereas a broad range of species from the order Trichoptera are known to have a low resistance to high salinity, although a few Trichoptera species, including the observed *E. tenellus*, exhibit salinity tolerance. Though *E. tenellus* was previously found in brackish water (4.3 g L^−1^) [71,94], in our study, two specimens of *E. tenellus* were found in the lower reach of the Ortozhina River with a salinity level of 16 g L^−1^. However, this finding must be interpreted with caution. The upper and middle reaches of the Otrozhina River are oligohaline; therefore, caddisflies *E. tenellus* could be brought to the polyhaline lower reach of this river by the current. In two rivers, the Solenaya Kuba and the Otrozhina rivers, the globally widely distributed brackish-water species *Gammarus lacustris*, have also been recorded with a salinity level of 16 g L^−1^, which is a new upper limit of salinity tolerance under natural conditions for this species. Earlier *Gammarus lacustris* was found in stream sections in which the highest salinity level was 11 g L^−1^ [95]. These findings correspond with those of several studies showing that Crustacea is the most salinity-tolerant group among the main invertebrate taxa [96,97].

The most salinity-resistant (halophilic) taxa observed in the investigated small rivers of the arid zone include the Chironomid species *Chironomus* sp., *Glyptotendipes salinus*, *Chironomus salinarius*, *Cricotopus salinophilus,* and *Tanytarsus kharaensis*, described for the first time in the rivers of the Lake Elton basin [98,99]. The larva and the pupa of the halophilic Ceratopogonid species *Palpomyia schmidti* also were described for the first time in the same area [100].

The study showed a link between the taxonomic richness of macrozoobenthic and aquatic salinity in small rivers, which supports our earlier findings of an overall decline in the taxonomic richness of macrozoobenthos in response to increasing salinity [73].

Under semi-arid and arid conditions, such as in the study area, the salinization of small rivers from oligohaline to mesohaline and polyhaline takes place under natural conditions. This leads to the development of depleted euryhaline and halophilic aquatic fauna. In particular, we consider that the five identified species (*Tanytarsus kharaensis*, *Glyptotendipes salinus*, *Cricotopus salinophilus*, *Chironomus salinarius*, and *Palpomyia schmidti*) with the highest salinity resistance can be used as indicators of salinization in aquatic ecosystems. Climate warming aggravated by anthropogenic impacts will intensify salinization processes in many arid regions of the world [6], which reduces the stability of already ecologically vulnerable natural ecosystems.

## 5. Conclusions

The findings demonstrate that the macrozoobenthic fauna of the 17 investigated small rivers in the arid southern part of the East European Plain is diverse (156 species), and it is predominantly represented by the lacustrine species from freshwater, euryhaline, and halophilic ecological groups.

The salinity gradient conditioned by the characteristics of the catchment basins, primarily the widespread occurrence of saline soils and groundwater, small channel gradients and slow-flowing or stagnant water bodies, and the aridization of climate conditions are the driving factors influencing the formation of macrozoobenthic communities. As a result, the species richness of the macrozoobenthic fauna is declining with an increase in aquatic salinity in these small rivers. The five identified halophilic species, i.e., *Tanytarsus kharaensis*, *Glyptotendipes salinus*, *Cricotopus salinophilus*, *Chironomus salinarius*, and *Palpomyia schmidti,* can be used as indicators of salinization in river ecosystems.

Follow-up research on the current analysis, which is based on the classical identification of species, should focus on analyzing the taxonomic richness of rivers with different salinity gradients using the eDNA method, which allows a more detailed analysis of the fauna and an expansion of the geographical scope.

## Figures and Tables

**Table 1:** Characteristics of the investigated small rivers in the southern arid region of the East European Plain.

No	River	Stream Order	Coordinates of the River Mouth, N, E	Catchment Area, km^2^	Length, km	Average Stream Slope, ‰	Current Velocity, m s^−1^	Salinity Min−Max, g L^−1^	Salinity Class	Salinity Average g L^−1^	Dissolved O_2_, mg L^−1^	pH
**Volga River basin**												
1	Solenaya Kuba	2	50°47′, 46°66′	2.03	98	0.56	0.005	0.38–3.96	hypohaline–oligohaline	1.94	8.7	8.01
2	Bizyuk	2	50°74′, 46°46′	0.71	54	1.08	0.005	0.27–0.43	hypohaline	0.33	6.5	8.28
3	Tarlyk	1	51°01′, 46°15′	0.63	51	1.59	0.03	0.27–0.61	hypohaline–oligohaline	0.42	4.6	8.1
4	Yama 1	2	50°19′, 46°26′	0.38	39	0.56	0.005	0.18–0.41	hypohaline	0.3	8.3	8.3
5	Zhidkaya Solyanka	2	50°79′, 47°04′	0.39	39	0.67	0.005	0.27–0.47	hypohaline	0.34	8.2	8.5
6	Kuba	2	50°19′, 46°25′	0.36	37	0.62	0.005	0.87–1.2	oligohaline	0.93	6.7	8.08
7	Vodyanka	2	50°12′, 47°12′	0.25	30	0.84	0.005	0.19–0.29	hypohaline	0.48	6.5	8.0
8	Otrozhina	2	50°46′, 46°73′	0.20	27	0.77	0.004	0.56–16	oligohalinemesohaline	7.8	9.0	8.8
9	Solyanka 2	3	50°76′, 46°97′	0.20	27	1.11	0.005	0.52–0.56	oligohaline	0.54	7.2	9.0
10	Yama	2	50°97′, 47°14′	0.13	21	0.75	0.005	0.27–0.39	hypohaline	0.35	8.1	8.06
11	Solyanka 3	2	50°13′, 46°20′	0.12	20	0.68	0.005	0.38–0.52	hypohaline–oligohaline	0.45	5.6	8.05
12	Gorkaya	2	50°35′, 46°54′	0.07	16	0.84	0.005	0.41–1.14	hypohaline–oligohaline	0.89	8.6	8.4
13	Kochetnaya	1	52°15′, 50°78′	0.06	14	1.97	0.01	0.53–1.1	oligohaline	0.82	7.7	7.0
14	Solyanka 1	2	50°47′, 46°53′	0.06	14	1.21	0.005	4.12–4.34	oligohaline	4.22	8.8	8.8
15	Gashon	2	50°97′, 46°91′	0.05	13	1.51	0.005	0.42–0.8	hypohaline–oligohaline	0.56	6.1	8.1
**Lake Elton basin**												
16	Solyanka	1	49°10′, 46°35′	0.018	6.7	5.52	0.12	28–30	polyhaline	28.7	12.6	7.5
17	Chernavka	1	49°12′, 46°40′	0.018	5.2	5.38	0.23	28–30	polyhaline	28.5	8.2	7.2

**Table 2:** Species composition of macrozoobenthos in the investigated small rivers.

Taxonomic Groups		Genus, Species
**Volga River basin**		
**Phylum Mollusca**	**Class Gastropoda**	*Anisus* sp., *Bithynia tentaculata* (Linnaeus, 1758), *Cincinna piscinalis* (Müller, 1774), *Cincinna* sp., *Lymnaea auricularia* (Linnaeus, 1758), *L. intermedia* (Lamark, 1822), *L. ovata* (Draparnaud, 1805), *Lymnaea* sp., *L. stagnalis* (Linnaeus, 1758), *Planorbis planorbis* (Linnaeus, 1758), and *Viviparus viviparus* (Linnaeus, 1758)
	**Class Bivalvia**	*Dreissena polymorpha* (Pallas, 1771), *Euglesa* sp., *Musculium* sp., and *Neopisidium* sp.
**Phylum Annelida** **Class Clitellata**	**Subclass Oligochaeta**	*Dero digitata* (Müller, 1773), *D. obtusa* Udekem, 1855, *Enchytraeus albidus* Henle, 1837, *Limnodrilus claparedeanus* Ratzel, 1868, *L. hoffmeisteri* Claparede, 1862, *L. udekemianus* Claparede, 1862, *Lumbriculus variegatus* (Müller, 1773), *Nais barbata* Müller, 1773, *N. communis* Piguet, 1906, *N. pardalis* Piquet, 1906, *N. pseudobtusa* Piguet, 1906, *N. variabilis* Piguet, 1906, *Ophidonais serpentina* (Müller, 1773), *Stylaria lacustris* (Linnaeus, 1767), *Tubifex tubifex* (Müller, 1773), and *Uncinais uncinata* (Oersted, 1842)
	**Order Hirudinea**	*Helobdella stagnalis* (Linnaeus, 1758), *Hemiclepsis marginata* (Müller, 1774) *Herpobdella octoculata* (Linnaeus, 1758), and *Piscicola geometra* (Linnaeus, 1761)
**Phylum Arthropoda**	**Subphylum Crustacea**	*Asellus aquaticus* (Linne, 1758), *Gammarus lacustris* Sars, 1863, *Chaetogammarus warpachowskyi* (Sars, 1894), *Katamysis warpachowskyi* G.O. Sars,1893, *Limnomysis benedeni* Czerniavsky, 1882, *Paramysis intermedia* (Czerniavsky, 1882), *P. lacustris* (Czerniavsky, 1882), and *Pterocuma rostrata* (G.O. Sars, 1894)
**Phylum Arthropoda** **Class Insecta**	**Order Odonata**	*Anax imperator* Leach, 1815, *Enallagma cyathigerum* Charpentier, 1840, *Erythromma najas* (Hansemann, 1823), *Ischnura elegans* Vanderlinden, 1823, *Lestes sponsa* (Hansemann, 1823), *Orthetrum cancellatum* (Linnaeus, 1758), *Sympecma fusca* (Vanderlinden., 1823), and *Sympetrum depressiusculum* (Sélys, 1841)
	**Order Ephemeroptera**	*Caenis robusta* (Eaton, 1884), *Cloeon* gr. *dipterum*, *C. simile* Eaton, 1870
	**Order Heteroptera**	*Cymatia coleoptrata* (Fabricius, 1777), *Gerris lacustris* (Linnaeus, 1758), *Hesperocorixa* sp., *Ilyocoris cimicoides* (Linnaeus, 1758), *Mesovelia furcata* Mulsant et Rey, 1852, *Micronecta* sp., *Microvelia* sp., *Notonecta glauca glauca* Linnaeus, 1758, *Plea minutissima* Leach, 1817, *Ranatra linearis* Linnaeus, 1758, and *Sigara* sp.
	**Order Coleoptera**	*Bagous argillaceus* Gyllenhal, 1836, *Berosus* sp., *Cybister* sp., *Donacia crassipes* Fabricius, 1775, *Haliplus ruficollis* (De Geer, 1774), *Haliplus* sp., *Helophorus paraminutus* Angus, 1986, *Hyphydrus ovatus* (Linnaeus, 1761), *Laccobius* sp., *Laccophilus* sp., *Noterus* clavicornis (De Geer, 1774), *Ochthebius* sp., *Paracymus aeneus* (Germar, 1824), *Peltodytes caesus* (Duftschmid, 1805), *Enochrus quadripunctatus* (Herbs, 1797), and *Hygrotus* sp.
	**Order Megaloptera**	*Sialis sordida* Klingstedt, 1932
	**Order Trichoptera**	*Agraylea multipunctata* Curtis, 1834, *Cyrnus flavidus* MacLachlan, 1864, *Ecnomus tenellus* (Rambur, 1842), *Hydroptila* sp., *Leptocerus tineiformis* Curtis, 1834, *Oecetis furva* (Rambur, 1842), and *Phryganea bipunctata* (Retzius, 1783)
	**Order Lepidoptera**	*Parapoynx stratiotata* Linnaeus, 1758
	**Order Diptera**	*Ablabesmyia monilis* (Linnaeus, 1758), *A. phatta* (Eggert, 1863), *Ablabesmyia* sp., *Anopheles* sp., *Bezzia* sp., *Chaoborus* sp., *Cricotopus* gr. *sylvestris*, *Chironomus melanescens* Keyl, 1961, *Ch. parathummi* Keyl, 1961, *Ch. plumosus* (Linnaeus, 1758), *Chironomus* sp., *Ch. salinarius* Kieffer 1915, *Cladopelma* gr. *lateralis*, *Cladotanytarsus mancus* (Walker, 1856), *Corynoneura coronata* Edwards, 1924, *C. scutellata* Winnertz, 1846, *Cricotopus caducus* Hirvenoja, 1973, *C. salinophilus* Zinchenko, Makarchenko et Makarchenko, 2009, *C*. gr. *sylvestris*, *Cricotopus* sp., *Cryptochironomus* gr. *defectus*, *Culicoides* sp., *Dasyhelea* sp., *Dicrotendipes nervosus* (Staeger, 1939), *D. notatus* (Meigen, 1818), *Endochironomus albipennis* (Meigen, 1830), *E. impar* (Walker, 1856), *Ephydra* sp., *Fleuria lacustris* Kieffer, 1924, *Glyptotendipes barbipes* (Staeger, 1839), *G. glaucus* (Meigen, 1818), *G*. *gripekoveni* (Kieffer, 1913), *G. paripes* Edwards, 1929, *G. salinus* Michailova, 1987, *Guttipelopia guttipennis* (Wulp, 1974), *Lauterborniella agrayloides* (Kieffer, 1911), *Macropelopia nebulosa* (Meigen, 1804), *Mallochohelea setigera* (Loew, 1864), *Mallochohelea* sp., *Microchironomus tener* (Kieffer, 1918), *Microtendipes pedellus* (de Geer, 1776), *Mochlonyx* sp., *Nanocladius bicolor* (Zetterstedt, 1838), *Odontomyia* sp., *Palpomyia* sp., *Palpomyia schmidti* Goetghebuer, 1934, *Paratanytarsus confusus* Palmen, 1960, *P.* gr. *lauterborni*, *Paratanytarsus* sp., *Parachironomus varus* Goetghebuer, 1921, *Podura aquatica* Linnaeus, 1758, *Polypedilum nubeculosum* (Meigen, 1804), *P. bicrenatum* Kieffer, 1921, *P. pedestre* (Meigen, 1830), *P. sordens* (van der Wulp, 1874), *Procladius ferrugineus* (Kieffer, 1918), *P. choreus* (Meigen, 1804), *Psectrocladius flavus* (Johannsen, 1905), *P. sordidellus* (Zetterstedt, 1838), *Sphaeromias pictus* (Meigen, 1818), *Stictochironomus crassiforceps* Kieffer, 1922, *S. rosenschöldi* (Zetterstedt, 1781), *Tanypus punctipennis* (Meigen, 1818), *Tanytarsus usmaënsis* Pagast, 1931, *T*. gr. *gregarius*, and *T. kharaensis* Zorina et Zinchenko, 2009
**Lake Elton basin**		
**Phylum Arthropoda** **Class Insecta**	**Order Heteroptera**	*Sigara* sp.
	**Order Coleoptera**	*Enochrus quadripunctatus* (Herbs, 1797), *Berosus* sp., *Hygrotus* sp.
	**Order Diptera**	*Chironomus salinarius* Kieffer 1915, *Cricotopus salinophilus* Zinchenko, Makarchenko et Makarchenko, 2009, *Ephydra* sp., *Palpomyia schmidti* Goetghebuer, 1934, and *Tanytarsus kharaensis* Zorina et Zinchenko, 2009

**Table 3:** Taxonomic structure of macrozoobenthos of the studied small rivers.

River	Ol *	Hi	Ml	Cr	Ep	Od	He	Tr	Co	Ch	Di	Others	In Total
**Volga River basin**													
Solenaya Kuba	9	3	6	2	2	2	6	6	1	29	6	-	72
Bizyuk	6	1	4	1	2	2	4	2	9	17	1	1	50
Tarlyk	9	3	8	6	2	1	3	3	1	25	2	2	65
Yama 1	5	-	1	1	2	1	2	-	1	15	2	-	30
Zhidkaya Solyanka	8	-	3	-	2	-	2	-	-	18	-	1	34
Kuba	5	-	-	1	3	1	1	2	2	16	3	1	35
Vodyanka	7	-	-	-	1	2	1	3	3	19	3	-	39
Otrozhina	5	2	2	2	2	1	5	3	2	27	4	1	56
Solyanka 2	2	-	-	1	1	1	1	-	1	13	3	1	24
Yama	7	1	1	1	2	1	2	-	3	18	1	-	37
Solyanka 3	3	-	-	1	-	1	-	-	2	14	2	-	23
Gorkaya	4	-	1	-	-	1	-	-	1	17	-	2	26
Kochetnaya	2	3	3	3	2	3	6	-	3	16	6	2	49
Solyanka 1	3	-	-	-	-	-	-	-	4	16	1	-	24
Gashon	3	-	-	-	2	-	2	-	-	12	-	-	19
**Lake Elton basin**													
Chernavka	-	-	-	-	-	-	1	-	3	2	2	-	8
Solyanka	-	-	-	-	-	-	1	-	1	2	2	-	6

* Ol—Oligochaeta, Hi—Hirudinea, Ml—Mollusca, Cr—Crustacea, Ep—Ephemeroptera, Od—Odonata, He—Heteroptera, Pl—Plecoptera, Tr—Trichoptera, Co—Coleoptera, Ch—Chironomidae, and Di—other Diptera; others—Arachnida, Megaloptera, and Lepidoptera.

**Table 4:** Eigenvalues, cumulative % variance, and significance of the CCA axes.

**Full Data Set**	**Axis 1**	**Axis 2**	**Axis 3**	**Axis 4**
Eigenvalues	0.900	0.218	0.216	0.169
Cumulative % variance of taxon data	32.8	40.8	48.6	54.8
Significance (probability) of axis	0.001	0.001	0.001	0.001
Sum of all unconstrained eigenvalues	2.743			
Sum of all canonical eigenvalues	2.126			
**Three Significant Variables**	**Axis 1**	**Axis 2**	**Axis 3**	**Axis 4**
Eigenvalues	0.891	0.193	0.165	0.149
Cumulative % variance of taxon data	32.5	39.5	45.6	51.0
Significance (probability) of axis	0.001	0.001	0.001	0.001
Sum of all unconstrained eigenvalues	2.743			
Sum of all canonical eigenvalues	1.494			

## Data Availability

The data presented in this study will be openly available in PANGEA upon article publication.
